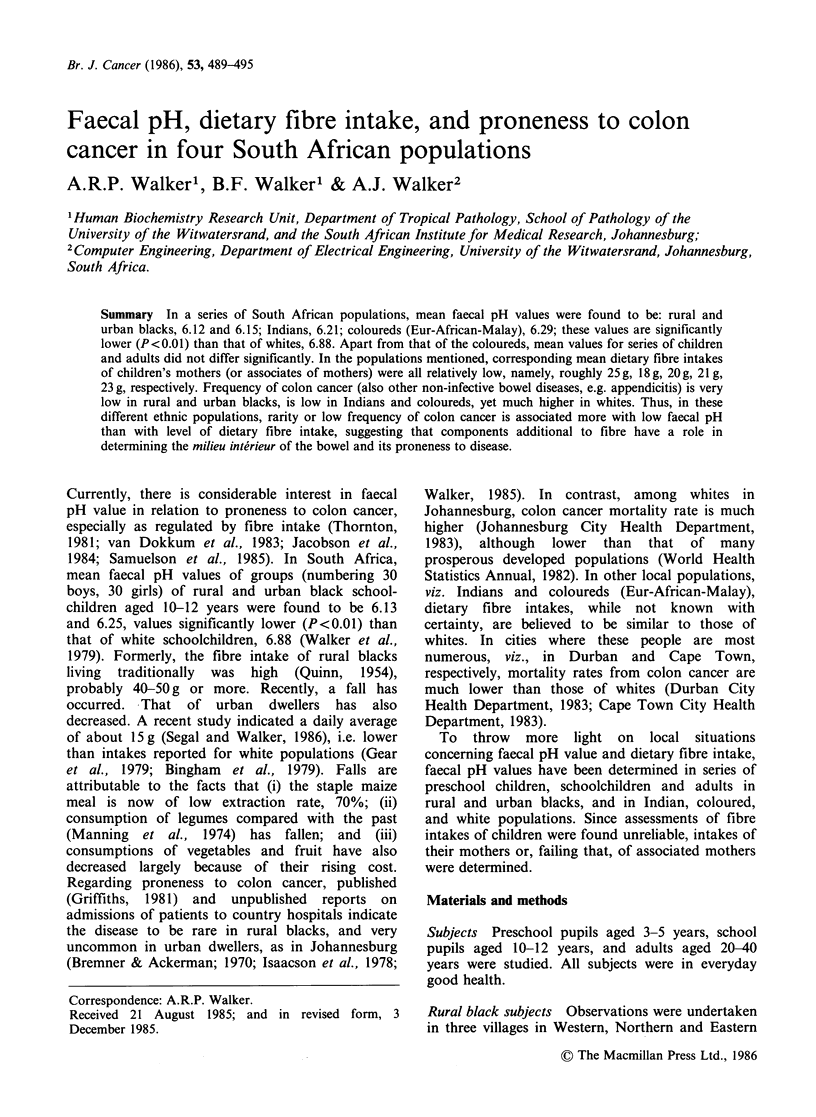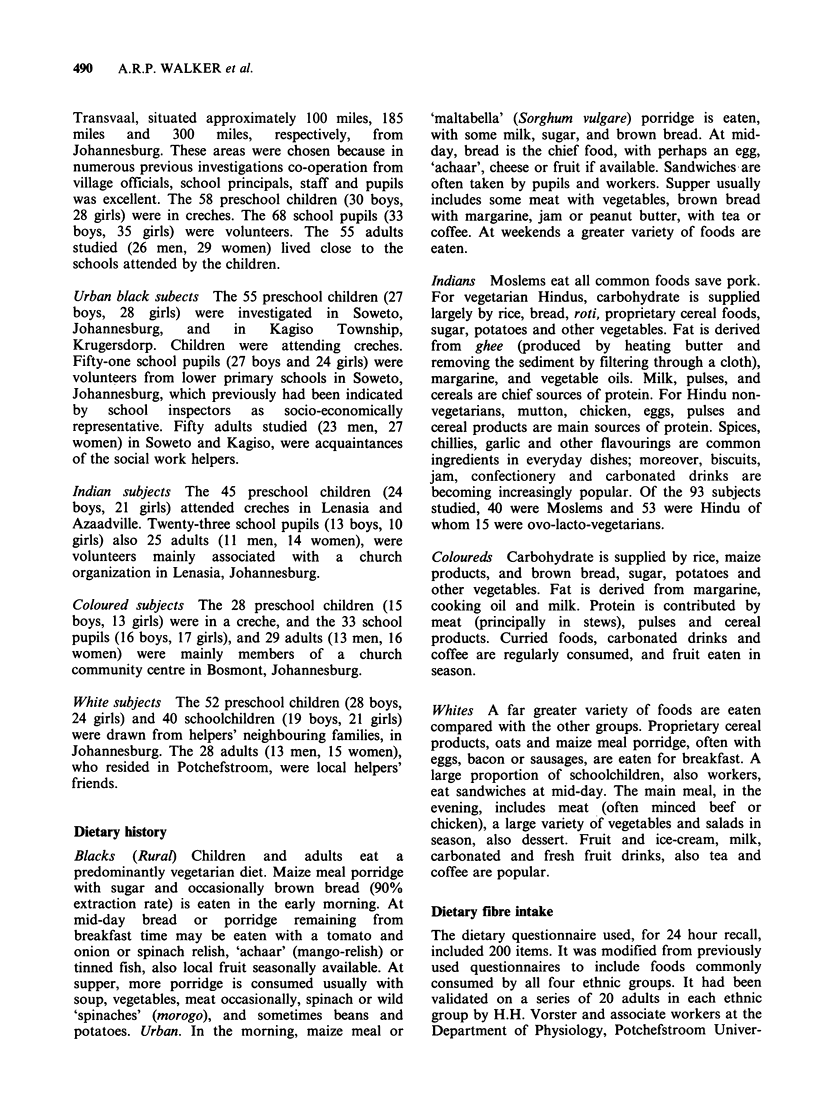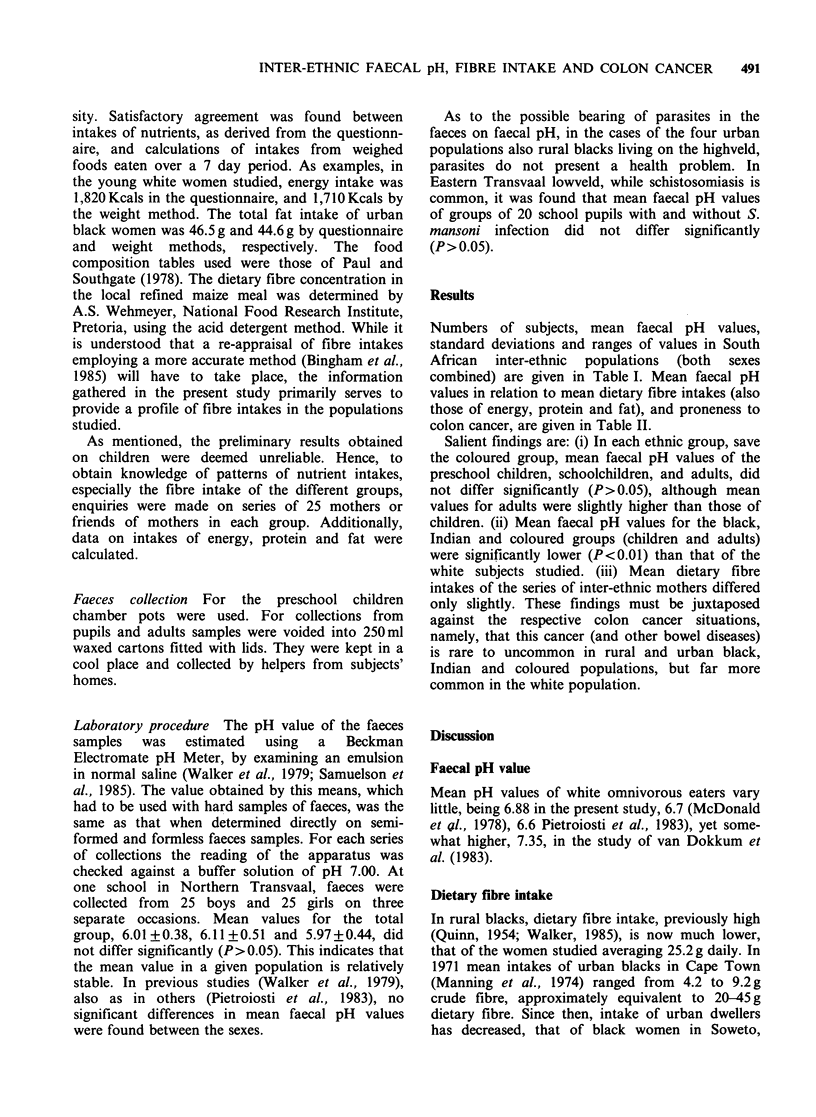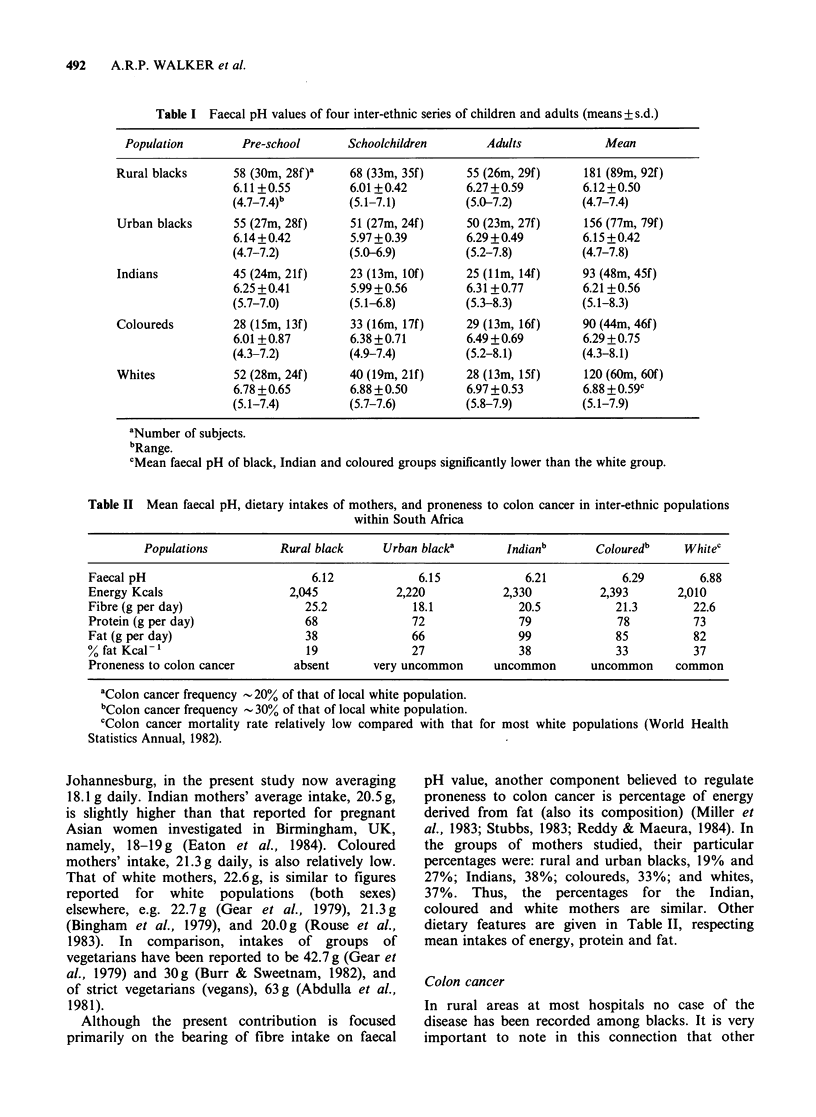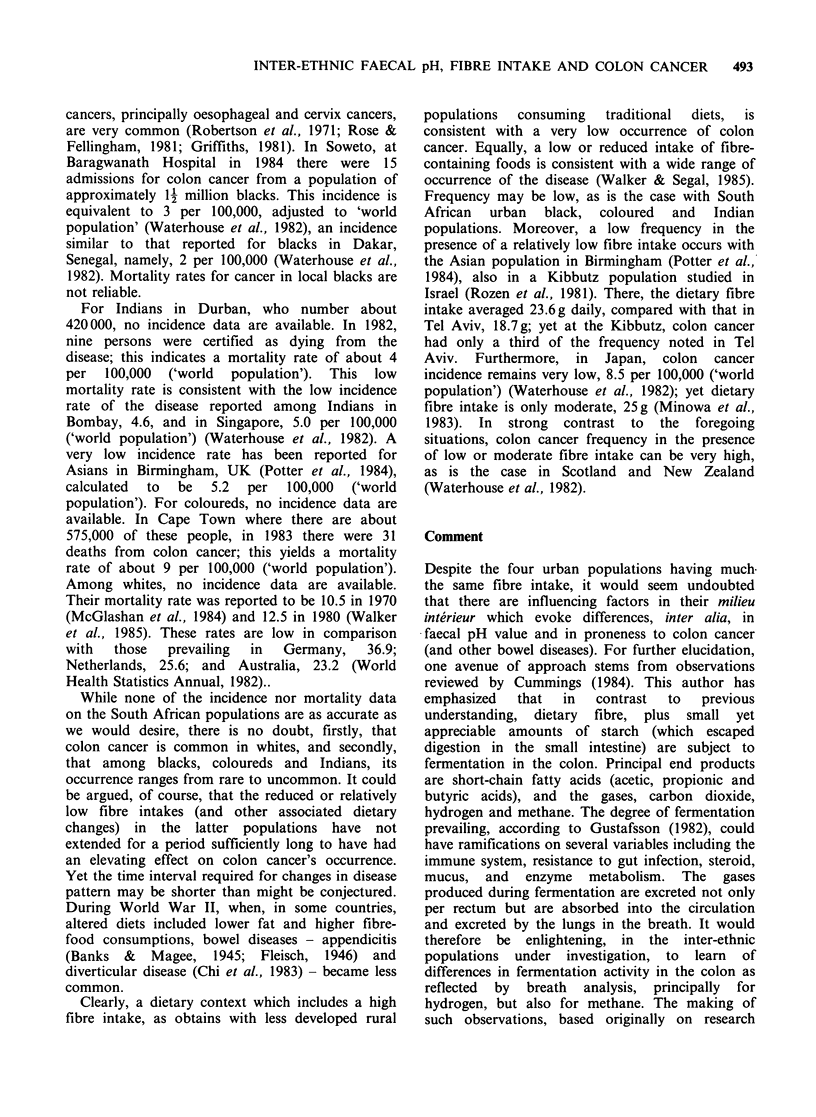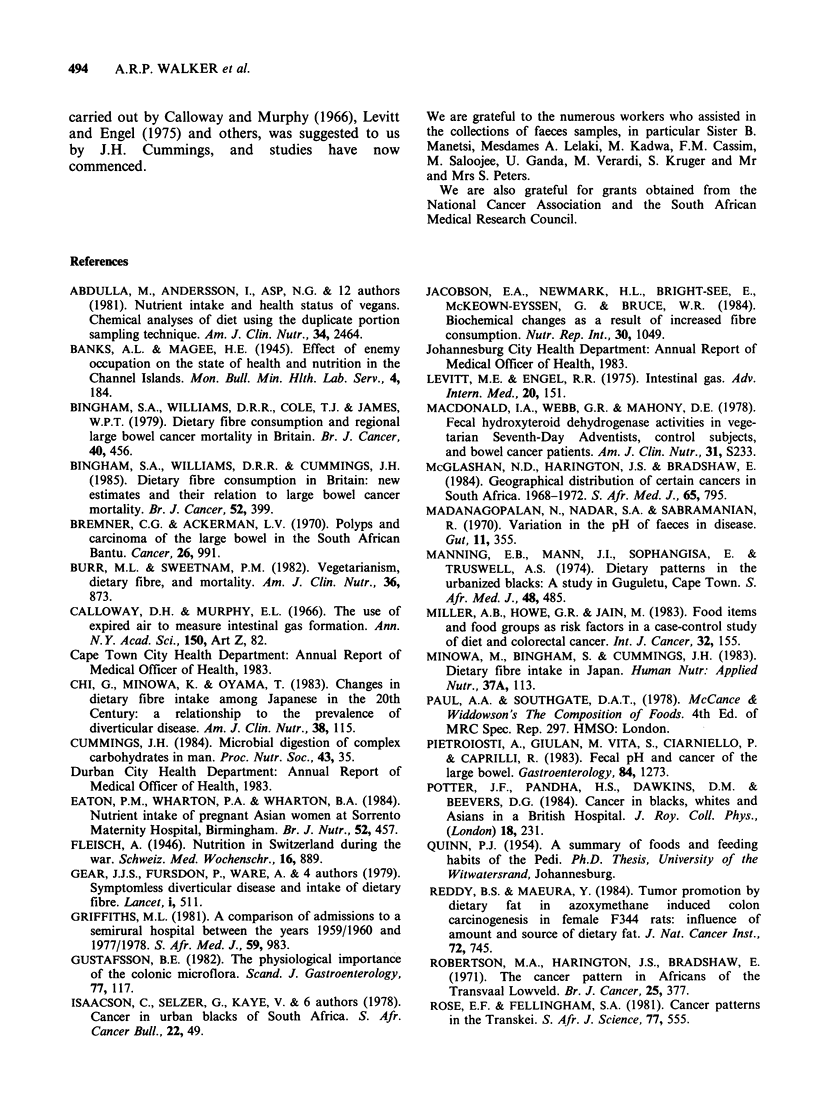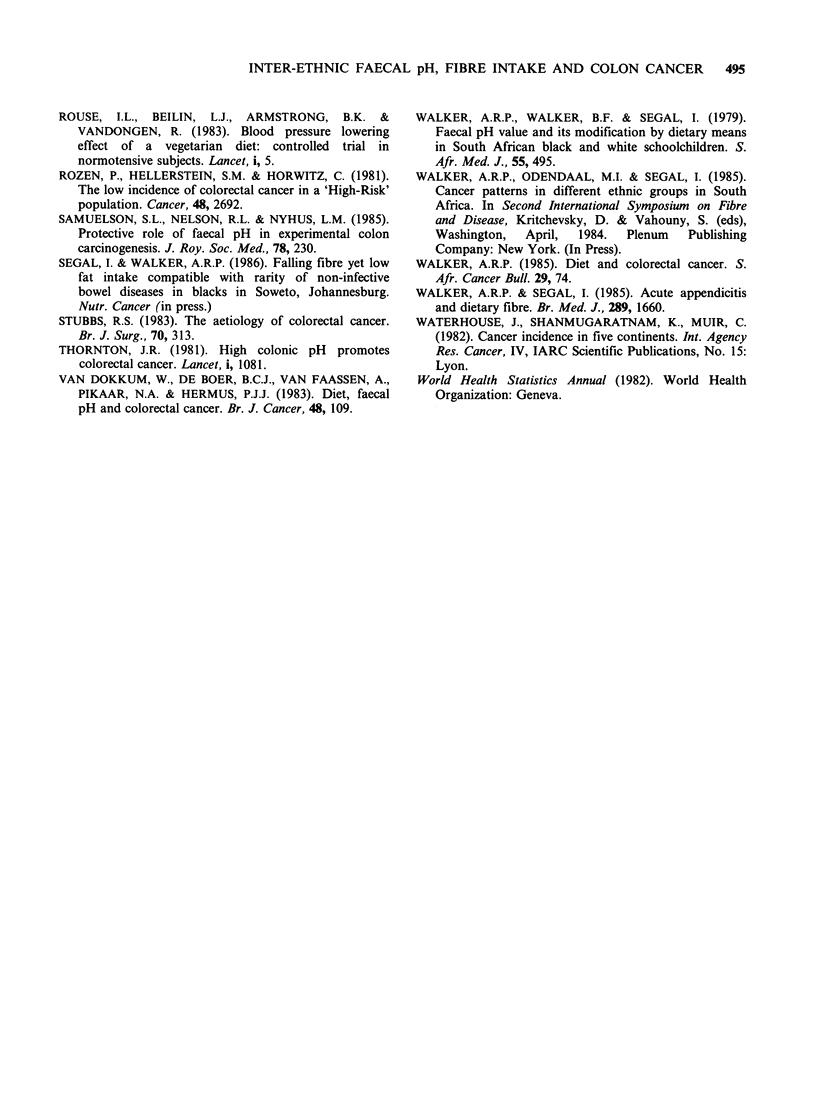# Faecal pH, dietary fibre intake, and proneness to colon cancer in four South African populations.

**DOI:** 10.1038/bjc.1986.77

**Published:** 1986-04

**Authors:** A. R. Walker, B. F. Walker, A. J. Walker

## Abstract

In a series of South African populations, mean faecal pH values were found to be: rural and urban blacks, 6.12 and 6.15; Indians 6.21; coloureds (Eur-African-Malay), 6.29; these are significantly lower (p less than 0.01) than that of whites, 6.88. Apart from that of the coloureds, mean values for series of children and adults did not differ significantly. In the populations mentioned, corresponding mean dietary fibre intakes of children's mothers (or associates of mothers) were all relatively low, namely, roughly 25 g, 18 g, 20 g, 21 g, 23 g, respectively. Frequency of colon cancer (also other non-infective bowel diseases, e.g. appendicitis) is very low in rural and urban blacks, is low in Indians and coloureds, yet much higher in whites. Thus, in these different ethnic populations, rarity or low frequency of colon cancer is associated more with low faecal pH than with level of dietary fibre intake, suggesting that components additional to fibre have a role in determining the milieu intérieur of the bowel and its proneness to disease.


*Br. J. Cancer* (1986), **53**, 489–495

### Faecal pH, dietary fibre intake, and proneness to colon cancer in four South African populations

A.R.P. Walker[1], B.F. Walker[1] & A.J. Walker[2]

[1]*Human Biochemistry Research Unit, Department of Tropical Pathology, School of Pathology of the University of the Witwatersrand, and the South African Institute for Medical Research, Johannesburg;* [2]*Computer Engineering, Department of Electrical Engineering, University of the Witwatersrand, Johannesburg, South Africa.*

**Summary** In a series of South African populations, mean faecal pH values were found to be: rural and urban blacks, 6.12 and 6.15; Indians, 6.21; coloureds (Eur-African-Malay), 6.29; these values are significantly lower ($P<0.01$) than that of whites, 6.88. Apart from that of the coloureds, mean values for series of children and adults did not differ significantly. In the populations mentioned, corresponding mean dietary fibre intakes of children's mothers (or associates of mothers) were all relatively low, namely, roughly 25 g, 18 g, 20 g, 21 g, 23 g, respectively. Frequency of colon cancer (also other non-infective bowel diseases, e.g. appendicitis) is very low in rural and urban blacks, is low in Indians and coloureds, yet much higher in whites. Thus, in these different ethnic populations, rarity or low frequency of colon cancer is associated more with low faecal pH than with level of dietary fibre intake, suggesting that components additional to fibre have a role in determining the *milieu intérieur* of the bowel and its proneness to disease.

Currently, there is considerable interest in faecal pH value in relation to proneness to colon cancer, especially as regulated by fibre intake (Thornton, 1981; van Dokkum *et al.*, 1983; Jacobson *et al.*, 1984; Samuelson *et al.*, 1985). In South Africa, mean faecal pH values of groups (numbering 30 boys, 30 girls) of rural and urban black schoolchildren aged 10–12 years were found to be 6.13 and 6.25, values significantly lower ($P<0.01$) than that of white schoolchildren, 6.88 (Walker *et al.*, 1979). Formerly, the fibre intake of rural blacks living traditionally was high (Quinn, 1954), probably 40–50 g or more. Recently, a fall has occurred. That of urban dwellers has also decreased. A recent study indicated a daily average of about 15 g (Segal and Walker, 1986), i.e. lower than intakes reported for white populations (Gear *et al.*, 1979; Bingham *et al.*, 1979). Falls are attributable to the facts that (i) the staple maize meal is now of low extraction rate, 70%; (ii) consumption of legumes compared with the past (Manning *et al.*, 1974) has fallen; and (iii) consumptions of vegetables and fruit have also decreased largely because of their rising cost. Regarding proneness to colon cancer, published (Griffiths, 1981) and unpublished reports on admissions of patients to country hospitals indicate the disease to be rare in rural blacks, and very uncommon in urban dwellers, as in Johannesburg (Bremner & Ackerman; 1970; Isaacson *et al.*, 1978; Walker, 1985). In contrast, among whites in Johannesburg, colon cancer mortality rate is much higher (Johannesburg City Health Department, 1983), although lower than that of many prosperous developed populations (World Health Statistics Annual, 1982). In other local populations, *viz.* Indians and coloureds (Eur-African-Malay), dietary fibre intakes, while not known with certainty, are believed to be similar to those of whites. In cities where these people are most numerous, *viz.*, in Durban and Cape Town, respectively, mortality rates from colon cancer are much lower than those of whites (Durban City Health Department, 1983; Cape Town City Health Department, 1983).

To throw more light on local situations concerning faecal pH value and dietary fibre intake, faecal pH values have been determined in series of preschool children, schoolchildren and adults in rural and urban blacks, and in Indian, coloured, and white populations. Since assessments of fibre intakes of children were found unreliable, intakes of their mothers or, failing that, of associated mothers were determined.

Correspondence: A.R.P. Walker.
Received 21 August 1985; and in revised form, 3 December 1985.

### Materials and methods

*Subjects* Preschool pupils aged 3–5 years, school pupils aged 10–12 years, and adults aged 20–40 years were studied. All subjects were in everyday good health.

*Rural black subjects* Observations were undertaken in three villages in Western, Northern and Eastern

© The Macmillan Press Ltd., 1986

Transvaal, situated approximately 100 miles, 185 miles and 300 miles, respectively, from Johannesburg. These areas were chosen because in numerous previous investigations co-operation from village officials, school principals, staff and pupils was excellent. The 58 preschool children (30 boys, 28 girls) were in creches. The 68 school pupils (33 boys, 35 girls) were volunteers. The 55 adults studied (26 men, 29 women) lived close to the schools attended by the children.

*Urban black subects* The 55 preschool children (27 boys, 28 girls) were investigated in Soweto, Johannesburg, and in Kagiso Township, Krugersdorp. Children were attending creches. Fifty-one school pupils (27 boys and 24 girls) were volunteers from lower primary schools in Soweto, Johannesburg, which previously had been indicated by school inspectors as socio-economically representative. Fifty adults studied (23 men, 27 women) in Soweto and Kagiso, were acquaintances of the social work helpers.

*Indian subjects* The 45 preschool children (24 boys, 21 girls) attended creches in Lenasia and Azaadville. Twenty-three school pupils (13 boys, 10 girls) also 25 adults (11 men, 14 women), were volunteers mainly associated with a church organization in Lenasia, Johannesburg.

*Coloured subjects* The 28 preschool children (15 boys, 13 girls) were in a creche, and the 33 school pupils (16 boys, 17 girls), and 29 adults (13 men, 16 women) were mainly members of a church community centre in Bosmont, Johannesburg.

*White subjects* The 52 preschool children (28 boys, 24 girls) and 40 schoolchildren (19 boys, 21 girls) were drawn from helpers' neighbouring families, in Johannesburg. The 28 adults (13 men, 15 women), who resided in Potchefstroom, were local helpers' friends.

**Dietary history**

*Blacks* (*Rural*) Children and adults eat a predominantly vegetarian diet. Maize meal porridge with sugar and occasionally brown bread (90% extraction rate) is eaten in the early morning. At mid-day bread or porridge remaining from breakfast time may be eaten with a tomato and onion or spinach relish, 'achaar' (mango-relish) or tinned fish, also local fruit seasonally available. At supper, more porridge is consumed usually with soup, vegetables, meat occasionally, spinach or wild 'spinaches' (*morogo*), and sometimes beans and potatoes. *Urban.* In the morning, maize meal or 'maltabella' (*Sorghum vulgare*) porridge is eaten, with some milk, sugar, and brown bread. At mid-day, bread is the chief food, with perhaps an egg, 'achaar', cheese or fruit if available. Sandwiches are often taken by pupils and workers. Supper usually includes some meat with vegetables, brown bread with margarine, jam or peanut butter, with tea or coffee. At weekends a greater variety of foods are eaten.

*Indians* Moslems eat all common foods save pork. For vegetarian Hindus, carbohydrate is supplied largely by rice, bread, *roti,* proprietary cereal foods, sugar, potatoes and other vegetables. Fat is derived from *ghee* (produced by heating butter and removing the sediment by filtering through a cloth), margarine, and vegetable oils. Milk, pulses, and cereals are chief sources of protein. For Hindu non-vegetarians, mutton, chicken, eggs, pulses and cereal products are main sources of protein. Spices, chillies, garlic and other flavourings are common ingredients in everyday dishes; moreover, biscuits, jam, confectionery and carbonated drinks are becoming increasingly popular. Of the 93 subjects studied, 40 were Moslems and 53 were Hindu of whom 15 were ovo-lacto-vegetarians.

*Coloureds* Carbohydrate is supplied by rice, maize products, and brown bread, sugar, potatoes and other vegetables. Fat is derived from margarine, cooking oil and milk. Protein is contributed by meat (principally in stews), pulses and cereal products. Curried foods, carbonated drinks and coffee are regularly consumed, and fruit eaten in season.

*Whites* A far greater variety of foods are eaten compared with the other groups. Proprietary cereal products, oats and maize meal porridge, often with eggs, bacon or sausages, are eaten for breakfast. A large proportion of schoolchildren, also workers, eat sandwiches at mid-day. The main meal, in the evening, includes meat (often minced beef or chicken), a large variety of vegetables and salads in season, also dessert. Fruit and ice-cream, milk, carbonated and fresh fruit drinks, also tea and coffee are popular.

**Dietary fibre intake**

The dietary questionnaire used, for 24 hour recall, included 200 items. It was modified from previously used questionnaires to include foods commonly consumed by all four ethnic groups. It had been validated on a series of 20 adults in each ethnic group by H.H. Vorster and associate workers at the Department of Physiology, Potchefstroom University. Satisfactory agreement was found between intakes of nutrients, as derived from the questionnaire, and calculations of intakes from weighed foods eaten over a 7 day period. As examples, in the young white women studied, energy intake was 1,820 Kcals in the questionnaire, and 1,710 Kcals by the weight method. The total fat intake of urban black women was 46.5 g and 44.6 g by questionnaire and weight methods, respectively. The food composition tables used were those of Paul and Southgate (1978). The dietary fibre concentration in the local refined maize meal was determined by A.S. Wehmeyer, National Food Research Institute, Pretoria, using the acid detergent method. While it is understood that a re-appraisal of fibre intakes employing a more accurate method (Bingham *et al.*, 1985) will have to take place, the information gathered in the present study primarily serves to provide a profile of fibre intakes in the populations studied.

As mentioned, the preliminary results obtained on children were deemed unreliable. Hence, to obtain knowledge of patterns of nutrient intakes, especially the fibre intake of the different groups, enquiries were made on series of 25 mothers or friends of mothers in each group. Additionally, data on intakes of energy, protein and fat were calculated.

*Faeces collection* For the preschool children chamber pots were used. For collections from pupils and adults samples were voided into 250 ml waxed cartons fitted with lids. They were kept in a cool place and collected by helpers from subjects' homes.

*Laboratory procedure* The pH value of the faeces samples was estimated using a Beckman Electromate pH Meter, by examining an emulsion in normal saline (Walker *et al.*, 1979; Samuelson *et al.*, 1985). The value obtained by this means, which had to be used with hard samples of faeces, was the same as that when determined directly on semiformed and formless faeces samples. For each series of collections the reading of the apparatus was checked against a buffer solution of pH 7.00. At one school in Northern Transvaal, faeces were collected from 25 boys and 25 girls on three separate occasions. Mean values for the total group, $6.01 \pm 0.38$, $6.11 \pm 0.51$ and $5.97 \pm 0.44$, did not differ significantly ($P > 0.05$). This indicates that the mean value in a given population is relatively stable. In previous studies (Walker *et al.*, 1979), also as in others (Pietroiosti *et al.*, 1983), no significant differences in mean faecal pH values were found between the sexes.

As to the possible bearing of parasites in the faeces on faecal pH, in the cases of the four urban populations also rural blacks living on the highveld, parasites do not present a health problem. In Eastern Transvaal lowveld, while schistosomiasis is common, it was found that mean faecal pH values of groups of 20 school pupils with and without *S. mansoni* infection did not differ significantly ($P > 0.05$).

### Results

Numbers of subjects, mean faecal pH values, standard deviations and ranges of values in South African inter-ethnic populations (both sexes combined) are given in Table I. Mean faecal pH values in relation to mean dietary fibre intakes (also those of energy, protein and fat), and proneness to colon cancer, are given in Table II.

Salient findings are: (i) In each ethnic group, save the coloured group, mean faecal pH values of the preschool children, schoolchildren, and adults, did not differ significantly ($P > 0.05$), although mean values for adults were slightly higher than those of children. (ii) Mean faecal pH values for the black, Indian and coloured groups (children and adults) were significantly lower ($P < 0.01$) than that of the white subjects studied. (iii) Mean dietary fibre intakes of the series of inter-ethnic mothers differed only slightly. These findings must be juxtaposed against the respective colon cancer situations, namely, that this cancer (and other bowel diseases) is rare to uncommon in rural and urban black, Indian and coloured populations, but far more common in the white population.

### Discussion

### Faecal pH value

Mean pH values of white omnivorous eaters vary little, being 6.88 in the present study, 6.7 (McDonald *et al.*, 1978), 6.6 Pietroiosti *et al.*, 1983), yet somewhat higher, 7.35, in the study of van Dokkum *et al.* (1983).

### Dietary fibre intake

In rural blacks, dietary fibre intake, previously high (Quinn, 1954; Walker, 1985), is now much lower, that of the women studied averaging 25.2 g daily. In 1971 mean intakes of urban blacks in Cape Town (Manning *et al.*, 1974) ranged from 4.2 to 9.2 g crude fibre, approximately equivalent to 20–45 g dietary fibre. Since then, intake of urban dwellers has decreased, that of black women in Soweto,

**Table I** Faecal pH values of four inter-ethnic series of children and adults (means ± s.d.)

| *Population* | *Pre-school* | *Schoolchildren* | *Adults* | *Mean* |
|---|---|---|---|---|
| Rural blacks | 58 (30m, 28f)[a]<br>6.11 ± 0.55<br>(4.7–7.4)[b] | 68 (33m, 35f)<br>6.01 ± 0.42<br>(5.1–7.1) | 55 (26m, 29f)<br>6.27 ± 0.59<br>(5.0–7.2) | 181 (89m, 92f)<br>6.12 ± 0.50<br>(4.7–7.4) |
| Urban blacks | 55 (27m, 28f)<br>6.14 ± 0.42<br>(4.7–7.2) | 51 (27m, 24f)<br>5.97 ± 0.39<br>(5.0–6.9) | 50 (23m, 27f)<br>6.29 ± 0.49<br>(5.2–7.8) | 156 (77m, 79f)<br>6.15 ± 0.42<br>(4.7–7.8) |
| Indians | 45 (24m, 21f)<br>6.25 ± 0.41<br>(5.7–7.0) | 23 (13m, 10f)<br>5.99 ± 0.56<br>(5.1–6.8) | 25 (11m, 14f)<br>6.31 ± 0.77<br>(5.3–8.3) | 93 (48m, 45f)<br>6.21 ± 0.56<br>(5.1–8.3) |
| Coloureds | 28 (15m, 13f)<br>6.01 ± 0.87<br>(4.3–7.2) | 33 (16m, 17f)<br>6.38 ± 0.71<br>(4.9–7.4) | 29 (13m, 16f)<br>6.49 ± 0.69<br>(5.2–8.1) | 90 (44m, 46f)<br>6.29 ± 0.75<br>(4.3–8.1) |
| Whites | 52 (28m, 24f)<br>6.78 ± 0.65<br>(5.1–7.4) | 40 (19m, 21f)<br>6.88 ± 0.50<br>(5.7–7.6) | 28 (13m, 15f)<br>6.97 ± 0.53<br>(5.8–7.9) | 120 (60m, 60f)<br>6.88 ± 0.59[c]<br>(5.1–7.9) |

[a]Number of subjects.
[b]Range.
[c]Mean faecal pH of black, Indian and coloured groups significantly lower than the white group.

**Table II** Mean faecal pH, dietary intakes of mothers, and proneness to colon cancer in inter-ethnic populations within South Africa

| *Populations* | *Rural black* | *Urban black*[a] | *Indian*[b] | *Coloured*[b] | *White*[c] |
|---|---|---|---|---|---|
| Faecal pH | 6.12 | 6.15 | 6.21 | 6.29 | 6.88 |
| Energy Kcals | 2,045 | 2,220 | 2,330 | 2,393 | 2,010 |
| Fibre (g per day) | 25.2 | 18.1 | 20.5 | 21.3 | 22.6 |
| Protein (g per day) | 68 | 72 | 79 | 78 | 73 |
| Fat (g per day) | 38 | 66 | 99 | 85 | 82 |
| % fat $Kcal^{-1}$ | 19 | 27 | 38 | 33 | 37 |
| Proneness to colon cancer | absent | very uncommon | uncommon | uncommon | common |

[a]Colon cancer frequency ~20% of that of local white population.
[b]Colon cancer frequency ~30% of that of local white population.
[c]Colon cancer mortality rate relatively low compared with that for most white populations (World Health Statistics Annual, 1982).

Johannesburg, in the present study now averaging 18.1 g daily. Indian mothers' average intake, 20.5 g, is slightly higher than that reported for pregnant Asian women investigated in Birmingham, UK, namely, 18–19 g (Eaton *et al.*, 1984). Coloured mothers' intake, 21.3 g daily, is also relatively low. That of white mothers, 22.6 g, is similar to figures reported for white populations (both sexes) elsewhere, e.g. 22.7 g (Gear *et al.*, 1979), 21.3 g (Bingham *et al.*, 1979), and 20.0 g (Rouse *et al.*, 1983). In comparison, intakes of groups of vegetarians have been reported to be 42.7 g (Gear *et al.*, 1979) and 30 g (Burr & Sweetnam, 1982), and of strict vegetarians (vegans), 63 g (Abdulla *et al.*, 1981).

Although the present contribution is focused primarily on the bearing of fibre intake on faecal pH value, another component believed to regulate proneness to colon cancer is percentage of energy derived from fat (also its composition) (Miller *et al.*, 1983; Stubbs, 1983; Reddy & Maeura, 1984). In the groups of mothers studied, their particular percentages were: rural and urban blacks, 19% and 27%; Indians, 38%; coloureds, 33%; and whites, 37%. Thus, the percentages for the Indian, coloured and white mothers are similar. Other dietary features are given in Table II, respecting mean intakes of energy, protein and fat.

### *Colon cancer*

In rural areas at most hospitals no case of the disease has been recorded among blacks. It is very important to note in this connection that other cancers, principally oesophageal and cervix cancers, are very common (Robertson *et al.*, 1971; Rose & Fellingham, 1981; Griffiths, 1981). In Soweto, at Baragwanath Hospital in 1984 there were 15 admissions for colon cancer from a population of approximately $1\frac{1}{2}$ million blacks. This incidence is equivalent to 3 per 100,000, adjusted to 'world population' (Waterhouse *et al.*, 1982), an incidence similar to that reported for blacks in Dakar, Senegal, namely, 2 per 100,000 (Waterhouse *et al.*, 1982). Mortality rates for cancer in local blacks are not reliable.

For Indians in Durban, who number about 420 000, no incidence data are available. In 1982, nine persons were certified as dying from the disease; this indicates a mortality rate of about 4 per 100,000 ('world population'). This low mortality rate is consistent with the low incidence rate of the disease reported among Indians in Bombay, 4.6, and in Singapore, 5.0 per 100,000 ('world population') (Waterhouse *et al.*, 1982). A very low incidence rate has been reported for Asians in Birmingham, UK (Potter *et al.*, 1984), calculated to be 5.2 per 100,000 ('world population'). For coloureds, no incidence data are available. In Cape Town where there are about 575,000 of these people, in 1983 there were 31 deaths from colon cancer; this yields a mortality rate of about 9 per 100,000 ('world population'). Among whites, no incidence data are available. Their mortality rate was reported to be 10.5 in 1970 (McGlashan *et al.*, 1984) and 12.5 in 1980 (Walker *et al.*, 1985). These rates are low in comparison with those prevailing in Germany, 36.9; Netherlands, 25.6; and Australia, 23.2 (World Health Statistics Annual, 1982)..

While none of the incidence nor mortality data on the South African populations are as accurate as we would desire, there is no doubt, firstly, that colon cancer is common in whites, and secondly, that among blacks, coloureds and Indians, its occurrence ranges from rare to uncommon. It could be argued, of course, that the reduced or relatively low fibre intakes (and other associated dietary changes) in the latter populations have not extended for a period sufficiently long to have had an elevating effect on colon cancer's occurrence. Yet the time interval required for changes in disease pattern may be shorter than might be conjectured. During World War II, when, in some countries, altered diets included lower fat and higher fibre-food consumptions, bowel diseases – appendicitis (Banks & Magee, 1945; Fleisch, 1946) and diverticular disease (Chi *et al.*, 1983) – became less common.

Clearly, a dietary context which includes a high fibre intake, as obtains with less developed rural populations consuming traditional diets, is consistent with a very low occurrence of colon cancer. Equally, a low or reduced intake of fibre-containing foods is consistent with a wide range of occurrence of the disease (Walker & Segal, 1985). Frequency may be low, as is the case with South African urban black, coloured and Indian populations. Moreover, a low frequency in the presence of a relatively low fibre intake occurs with the Asian population in Birmingham (Potter *et al.*, 1984), also in a Kibbutz population studied in Israel (Rozen *et al.*, 1981). There, the dietary fibre intake averaged 23.6 g daily, compared with that in Tel Aviv, 18.7 g; yet at the Kibbutz, colon cancer had only a third of the frequency noted in Tel Aviv. Furthermore, in Japan, colon cancer incidence remains very low, 8.5 per 100,000 ('world population') (Waterhouse *et al.*, 1982); yet dietary fibre intake is only moderate, 25 g (Minowa *et al.*, 1983). In strong contrast to the foregoing situations, colon cancer frequency in the presence of low or moderate fibre intake can be very high, as is the case in Scotland and New Zealand (Waterhouse *et al.*, 1982).

### Comment

Despite the four urban populations having much the same fibre intake, it would seem undoubted that there are influencing factors in their *milieu intérieur* which evoke differences, *inter alia*, in faecal pH value and in proneness to colon cancer (and other bowel diseases). For further elucidation, one avenue of approach stems from observations reviewed by Cummings (1984). This author has emphasized that in contrast to previous understanding, dietary fibre, plus small yet appreciable amounts of starch (which escaped digestion in the small intestine) are subject to fermentation in the colon. Principal end products are short-chain fatty acids (acetic, propionic and butyric acids), and the gases, carbon dioxide, hydrogen and methane. The degree of fermentation prevailing, according to Gustafsson (1982), could have ramifications on several variables including the immune system, resistance to gut infection, steroid, mucus, and enzyme metabolism. The gases produced during fermentation are excreted not only per rectum but are absorbed into the circulation and excreted by the lungs in the breath. It would therefore be enlightening, in the inter-ethnic populations under investigation, to learn of differences in fermentation activity in the colon as reflected by breath analysis, principally for hydrogen, but also for methane. The making of such observations, based originally on research carried out by Calloway and Murphy (1966), Levitt and Engel (1975) and others, was suggested to us by J.H. Cummings, and studies have now commenced.

We are grateful to the numerous workers who assisted in the collections of faeces samples, in particular Sister B. Manetsi, Mesdames A. Lelaki, M. Kadwa, F.M. Cassim, M. Saloojee, U. Ganda, M. Verardi, S. Kruger and Mr and Mrs S. Peters.

We are also grateful for grants obtained from the National Cancer Association and the South African Medical Research Council.

### References

ABDULLA, M., ANDERSSON, I., ASP, N.G. & 12 authors (1981). Nutrient intake and health status of vegans. Chemical analyses of diet using the duplicate portion sampling technique. *Am. J. Clin. Nutr.*, **34**, 2464.

BANKS, A.L. & MAGEE, H.E. (1945). Effect of enemy occupation on the state of health and nutrition in the Channel Islands. *Mon. Bull. Min. Hlth. Lab. Serv.*, **4**, 184.

BINGHAM, S.A., WILLIAMS, D.R.R., COLE, T.J. & JAMES, W.P.T. (1979). Dietary fibre consumption and regional large bowel cancer mortality in Britain. *Br. J. Cancer*, **40**, 456.

BINGHAM, S.A., WILLIAMS, D.R.R. & CUMMINGS, J.H. (1985). Dietary fibre consumption in Britain: new estimates and their relation to large bowel cancer mortality. *Br. J. Cancer*, **52**, 399.

BREMNER, C.G. & ACKERMAN, L.V. (1970). Polyps and carcinoma of the large bowel in the South African Bantu. *Cancer*, **26**, 991.

BURR, M.L. & SWEETNAM, P.M. (1982). Vegetarianism, dietary fibre, and mortality. *Am. J. Clin. Nutr.*, **36**, 873.

CALLOWAY, D.H. & MURPHY, E.L. (1966). The use of expired air to measure intestinal gas formation. *Ann. N.Y. Acad. Sci.*, **150**, Art Z, 82.

Cape Town City Health Department: Annual Report of Medical Officer of Health, 1983.

CHI, G., MINOWA, K. & OYAMA, T. (1983). Changes in dietary fibre intake among Japanese in the 20th Century: a relationship to the prevalence of diverticular disease. *Am. J. Clin. Nutr.*, **38**, 115.

CUMMINGS, J.H. (1984). Microbial digestion of complex carbohydrates in man. *Proc. Nutr. Soc.*, **43**, 35.

Durban City Health Department: Annual Report of Medical Officer of Health, 1983.

EATON, P.M., WHARTON, P.A. & WHARTON, B.A. (1984). Nutrient intake of pregnant Asian women at Sorrento Maternity Hospital, Birmingham. *Br. J. Nutr.*, **52**, 457.

FLEISCH, A. (1946). Nutrition in Switzerland during the war. *Schweiz. Med. Wochenschr.*, **16**, 889.

GEAR, J.J.S., FURSDON, P., WARE, A. & 4 authors (1979). Symptomless diverticular disease and intake of dietary fibre. *Lancet*, **i**, 511.

GRIFFITHS, M.L. (1981). A comparison of admissions to a semirural hospital between the years 1959/1960 and 1977/1978. *S. Afr. Med. J.*, **59**, 983.

GUSTAFSSON, B.E. (1982). The physiological importance of the colonic microflora. *Scand. J. Gastroenterology*, **77**, 117.

ISAACSON, C., SELZER, G., KAYE, V. & 6 authors (1978). Cancer in urban blacks of South Africa. *S. Afr. Cancer Bull.*, **22**, 49.

JACOBSON, E.A., NEWMARK, H.L., BRIGHT-SEE, E., McKEOWN-EYSSEN, G. & BRUCE, W.R. (1984). Biochemical changes as a result of increased fibre consumption. *Nutr. Rep. Int.*, **30**, 1049.

Johannesburg City Health Department: Annual Report of Medical Officer of Health, 1983.

LEVITT, M.E. & ENGEL, R.R. (1975). Intestinal gas. *Adv. Intern. Med.*, **20**, 151.

MACDONALD, I.A., WEBB, G.R. & MAHONY, D.E. (1978). Fecal hydroxyteroid dehydrogenase activities in vegetarian Seventh-Day Adventists, control subjects, and bowel cancer patients. *Am. J. Clin. Nutr.*, **31**, S233.

McGLASHAN, N.D., HARINGTON, J.S. & BRADSHAW, E. (1984). Geographical distribution of certain cancers in South Africa. 1968–1972. *S. Afr. Med. J.*, **65**, 795.

MADANAGOPALAN, N., NADAR, S.A. & SABRAMANIAN, R. (1970). Variation in the pH of faeces in disease. *Gut*, **11**, 355.

MANNING, E.B., MANN, J.I., SOPHANGISA, E. & TRUSWELL, A.S. (1974). Dietary patterns in the urbanized blacks: A study in Guguletu, Cape Town. *S. Afr. Med. J.*, **48**, 485.

MILLER, A.B., HOWE, G.R. & JAIN, M. (1983). Food items and food groups as risk factors in a case-control study of diet and colorectal cancer. *Int. J. Cancer*, **32**, 155.

MINOWA, M., BINGHAM, S. & CUMMINGS, J.H. (1983). Dietary fibre intake in Japan. *Human Nutr: Applied Nutr.*, **37A**, 113.

PAUL, A.A. & SOUTHGATE, D.A.T., (1978). *McCance & Widdowson's The Composition of Foods*. 4th Ed. of MRC Spec. Rep. 297. HMSO: London.

PIETROIOSTI, A., GIULAN, M. VITA, S., CIARNIELLO, P. & CAPRILLI, R. (1983). Fecal pH and cancer of the large bowel. *Gastroenterology*, **84**, 1273.

POTTER, J.F., PANDHA, H.S., DAWKINS, D.M. & BEEVERS, D.G. (1984). Cancer in blacks, whites and Asians in a British Hospital. *J. Roy. Coll. Phys., (London)* **18**, 231.

QUINN, P.J. (1954). A summary of foods and feeding habits of the Pedi. *Ph.D. Thesis, University of the Witwatersrand*, Johannesburg.

REDDY, B.S. & MAEURA, Y. (1984). Tumor promotion by dietary fat in azoxymethane induced colon carcinogenesis in female F344 rats: influence of amount and source of dietary fat. *J. Nat. Cancer Inst.*, **72**, 745.

ROBERTSON, M.A., HARINGTON, J.S., BRADSHAW, E. (1971). The cancer pattern in Africans of the Transvaal Lowveld. *Br. J. Cancer*, **25**, 377.

ROSE, E.F. & FELLINGHAM, S.A. (1981). Cancer patterns in the Transkei. *S. Afr. J. Science*, **77**, 555.

ROUSE, I.L., BEILIN, L.J., ARMSTRONG, B.K. & VANDONGEN, R. (1983). Blood pressure lowering effect of a vegetarian diet: controlled trial in normotensive subjects. *Lancet*, **i**, 5.

ROZEN, P., HELLERSTEIN, S.M. & HORWITZ, C. (1981). The low incidence of colorectal cancer in a 'High-Risk' population. *Cancer*, **48**, 2692.

SAMUELSON, S.L., NELSON, R.L. & NYHUS, L.M. (1985). Protective role of faecal pH in experimental colon carcinogenesis. *J. Roy. Soc. Med.*, **78**, 230.

SEGAL, I. & WALKER, A.R.P. (1986). Falling fibre yet low fat intake compatible with rarity of non-infective bowel diseases in blacks in Soweto, Johannesburg. *Nutr. Cancer* (in press.)

STUBBS, R.S. (1983). The aetiology of colorectal cancer. *Br. J. Surg.*, **70**, 313.

THORNTON, J.R. (1981). High colonic pH promotes colorectal cancer. *Lancet*, **i**, 1081.

VAN DOKKUM, W., DE BOER, B.C.J., VAN FAASSEN, A., PIKAAR, N.A. & HERMUS, P.J.J. (1983). Diet, faecal pH and colorectal cancer. *Br. J. Cancer*, **48**, 109.

WALKER, A.R.P., WALKER, B.F. & SEGAL, I. (1979). Faecal pH value and its modification by dietary means in South African black and white schoolchildren. *S. Afr. Med. J.*, **55**, 495.

WALKER, A.R.P., ODENDAAL, M.I. & SEGAL, I. (1985). Cancer patterns in different ethnic groups in South Africa. In *Second International Symposium on Fibre and Disease*, Kritchevsky, D. & Vahouny, S. (eds), Washington, April, 1984. Plenum Publishing Company: New York. (In Press).

WALKER, A.R.P. (1985). Diet and colorectal cancer. *S. Afr. Cancer Bull.* **29**, 74.

WALKER, A.R.P. & SEGAL, I. (1985). Acute appendicitis and dietary fibre. *Br. Med. J.*, **289**, 1660.

WATERHOUSE, J., SHANMUGARATNAM, K., MUIR, C. (1982). Cancer incidence in five continents. *Int. Agency Res. Cancer*, IV, IARC Scientific Publications, No. 15: Lyon.

*World Health Statistics Annual* (1982). World Health Organization: Geneva.